# A new cryptic species of green pit viper of the genus *Trimeresurus* Lacépède, 1804 (Serpentes, Viperidae) from northeast India

**DOI:** 10.1371/journal.pone.0268402

**Published:** 2022-05-20

**Authors:** Yashpal Singh Rathee, Jayaditya Purkayastha, Hmar Tlawmte Lalremsanga, Siddharth Dalal, Lal Biakzuala, Lal Muansanga, Zeeshan A. Mirza

**Affiliations:** 1 Herpsmitten, Umroi Military Station, Umiam, Meghalaya, India; 2 Help Earth, Raghunath Choudhury Path, Lachitnagar, Guwahati, Assam, India; 3 Department of Zoology, Mizoram University, Aizawl, Mizoram, India; 4 National Centre for Biological Sciences, TIFR, Bangalore, Karnataka, India; Guangxi University, CHINA

## Abstract

A new cryptic species of green pit viper is described from northeast India, based on specimens collected from the state of Mizoram and Meghalaya. The new species is a member of the subgenus *Viridovipera* and is sister to *Trimeresurus medoensis* based on molecular data for mitochondrial cytochrome b gene, whereas resembles *Trimeresurus gumprechti* morphologically. A combination of characters helps delimit the new species from its congeners. Description of the new species highlights the need for dedicated surveys across northeast India to document its reptilian diversity, as this represents the third new species of the genus to be described in the past three years.

## Introduction

The genus *Trimeresurus* Lacépède 1804 currently comprises 43 nominate species [1] of venomous terrestrial to arboreal species distributed across south and southeast Asia. Of these the *T*. *albolabris* Gray, 1842, *T*. *arunachalensis* Captain, Deepak, Pandit, Bhatt & Athreya, 2019 *T*. *erythrurus* (Cantor, 1839), *T*. *gumprechti*, David, Vogel, Pauwels & Vidal, 2002, *T*. *medoensis* Zhao, 1977, *T*. *popeorum* Smith, 1937, *T*. *salazar* Mirza, Bhosale, Phansalkar, Sawant Gowande, Patel 2020, *T*. *septentrionalis* Kramer, 1977, and *T*. *yunnanensis* Schmidt, 1925, are recorded from Northeast India [2]. Though the above species are recorded from Northeast India, there still exists confusion related to presence of some of these species from the region. Apparent morphological similarities between species and cryptic diversity within the genus *Trimeresurus* often result in misidentification [3–6]. For example, recent work on the herpetofauna of the region [7, 8] reported *T*. *albolabris* from several localities across the region [9, 10] most likely to be attributed to be *T*. *salazar* as highlighted by Rathee et al. [11] & Malik et al. [12].

A similar scenario appears to be the case with the records of *T*. *gumprechti* (see discussion), which has been recorded from the state of Meghalaya [13]. Recent collection of specimens from the state and re-examination of museum specimens enabled us to ascertain the identity of the population currently referred to either of the following species: *T*. *yunnanensis*, *T*. *stejnegeri* and T. *gumprechti* in India based on molecular as well as morphological data. Results from morphological and molecular data analysis support the distinctness of the species from the aforementioned species and is herein described as a new species.

## Material and methods

### Morphology

The study was conducted under permit no. FWC/G/173/Pt-V/2377-87 and A.33011/2/99- CWLW/225 issued by the Environment, Forests & Climate Change Department, Government of Meghalaya and Environment, Forests & Climate Change Department, Government Mizoram, respectively. Six specimens of the new species were collected by hand in the field, photographed, and later euthanized with halothane within 24 h of capture following ethical guidelines for animal euthanasia [14]. The specimens were fixed in 8% formaldehyde buffer and later stored in 70% ethanol. Liver tissue was collected for molecular work and stored in molecular grade ethanol prior to specimen fixation. The specimens have been deposited in the collection of the research collection facility of the National Centre for Biological Sciences, Bangalore, Bombay Natural History Society (BNHS), Mumbai and Zoological Survey of India, ERS, Shillong. Measurements were taken with the help of digital callipers to the nearest 0.1 mm and those for snout to vent length (SVL) and tail length (TaL) were taken with a string, which was then measured using a scale. Ventral scales (V) were counted as directed by Dowling [15]. Dorsal scales at midbody were counted at midway of the SVL. Cephalic scales (CEP) number was counted on a straight line between the middle of the supraoculars; longitudinal cephalic scales (LCS) number of scales counted from the posterior border off the internasals to the neck (which is here defined as the dorsal scale row, which corresponds to the first ventral scale). Abbreviations used in the description: TL = total length, HL = head length measured from snout tip to the angle of the jaw, DEL = distance between lower eye margin and the edge of lip. Morphological data for the new species were compared with the types of the sister taxa based on molecular data and original descriptions of sister species were referred too. Morphological data from literature were derived from Gumprecht et al. [9], Kramer [16], David et al. [17] and Regenass and Kramer [18]. MicroCT scans were generated following protocols outlined in Mirza et al. [2]. Skull features and terminologies follow Cundall and Irish [19].

### Institution acronyms

BNHS = Bombay Natural History Society, Mumbai, INDIA; MNHN = Muséum national d’Histoire naturelle, Paris, FRANCE; MZMU = Departmental Museum of Zoology, Mizoram University, Aizawl, INDIA; NCBS = Collection Facility of the National Centre for Biological Sciences, Bangalore, INDIA; NHM = Natural History Museum, London, UK; ZMUC = Zoological Museum University of Copenhagen, DENMARK; ZSI/ERS = Zoological Survey of India, Eastern Region Station, Shillong, INDIA.

Comparative material examined:

*Trimeresurus albolabris* NHMUK 1946.1.19.85, lectoype, China; BNHS 2652–2653, Taungyi Myanmar; BNHS 2654, Mayniyo, Myanmar; BNHS 2655, Myanmar; BNHS 2656, Bangkok, Thailand; BNHS 2659, Moulinein, Myanmar; BNHS 3304, Car Nicobar, India

*Trimeresurus davidi* NHMUK 1936.7.7.40, NHMUK 1936.7.7.41, NHMUK 1936.7.7.42, paratypes, adult females from ‘Car Nicobar, Nicobar Islands, India

*Trimeresurus erythrurus* NCBS-AG767, NCBS-AG776, NCBS-AG781–782, Tripura, India

*Trimeresurus gumprechti* MNHN 1999.9072 holotype & MNHN 1999.9073 paratype, Loei, Loei Province, Thailand, paratype

*Trimeresurus popeiorum* NHMUK 1872.4.17.137, lectotype, Khasi hills, Meghalaya, India

*Trimeresurus salazar* BNHS 3554, holotype, Pakke Tiger Reserve, Arunachal Pradesh, India, ZMUC R69255-R69256 Assam, India

*Trimeresurus septentrionalis* NHMUK 1937.3.1.15 paratype, Kullu District, Himachal Pradesh, India; paratype NHMUK 1937.3.1.15, paratype NHMUK 1955.1.13.82

### Nomenclature acts

The electronic edition of this article conforms to the requirements of the amended International Code of Zoological Nomenclature, and hence the new names contained herein are available under that Code from the electronic edition of this article. This published work and the nomenclatural acts it contains have been registered in ZooBank, the online registration system for the ICZN. The ZooBank LSIDs (Life Science Identifiers) can be resolved and the associated information viewed through any standard web browser by appending the LSID to the prefix “http://zoobank.org/”. The LSID for this publication is: urn:lsid:zoobank.org:pub:18F10D56-F31B-4D51-ADC4-B793256075D2. The electronic edition of this work was published in a journal with an ISSN, and has been archived and is available from the following digital repositories: LOCKSS [author to insert any additional repositories].

### Molecular analysis

Genomic DNA was isolated from the preserved tissues of the type specimens using QIAGEN DNeasy kits, following protocols directed by the manufacturer. Molecular methods follow Mirza et al. [20] and Mirza and Patel [21]. A fragment of the mitochondrial cytochrome b (*cyt* b) gene was amplified using primers used by Pyron et al. [22] and Mirza et al. [20]. A 22.4 μl reaction was set for a bi-directional Polymerase Chain Reaction (PCR), containing 10 μl of Thermo Scientific DreamTaq PCR Master Mix, 10 μl of molecular grade water, 0.2 μl of each 10 μM primer and 2 μl template DNA, carried out with an Applied Biosystems ProFlex PCR System. Thermo-cycle profile used for amplification were as follows: 95°C for 3 min, (denaturation temperature 95°C for 30 sec, annealing temperature 48°C for 45 sec, elongation temperature 72°C for 1 minutes) × 36 cycles, 72°C for 10 min, hold at 4°C. PCR product was cleaned using QIAquick PCR Purification Kit and sequenced with an Applied Biosystems 3730 DNA Analyzer. Besides this, sequences of *Trimeresurus* spp. available on GenBank® were downloaded for calculating genetic divergence and/or molecular phylogenetic reconstructions. Taxa for molecular phylogenetics through a Maximum Likelihood approach, were selected based on the tree topologies recovered by Mirza et al. [2]. Sequences were aligned in MegaX [23] using ClustalW [24] with default settings. The aligned dataset was subject to Maximum Likelihood (ML) phylogenetics on the IQ-TREE (http://iqtree.cibiv.univie.ac.at/) online portal [25]. Sequence substitution model was selected using the auto parameter with provision for FreeRate heterogeneity. The analysis was run with an ultrafast bootstrap option for 1000 iterations to assess clade support. Bayesian Inference (BI) was executed through MrBayes [26]. Generalised time reversible + Gamma model of sequence substitution for 10 million generations sampled every 1000^th^ generation. The analysis was terminated after the run reached a split frequency below 0.01 and convergence was assessed by checking if the ESS values were greater than 200 for all parameters using Tracer v1.6. First twenty-five percent of the trees were discarded as burn-in. Uncorrected pairwise *p*-distance (% sequence divergence) was calculated in MegaX with pairwise deletions of missing data and gaps.

## Results

### Molecular analysis

**Fig 1:**
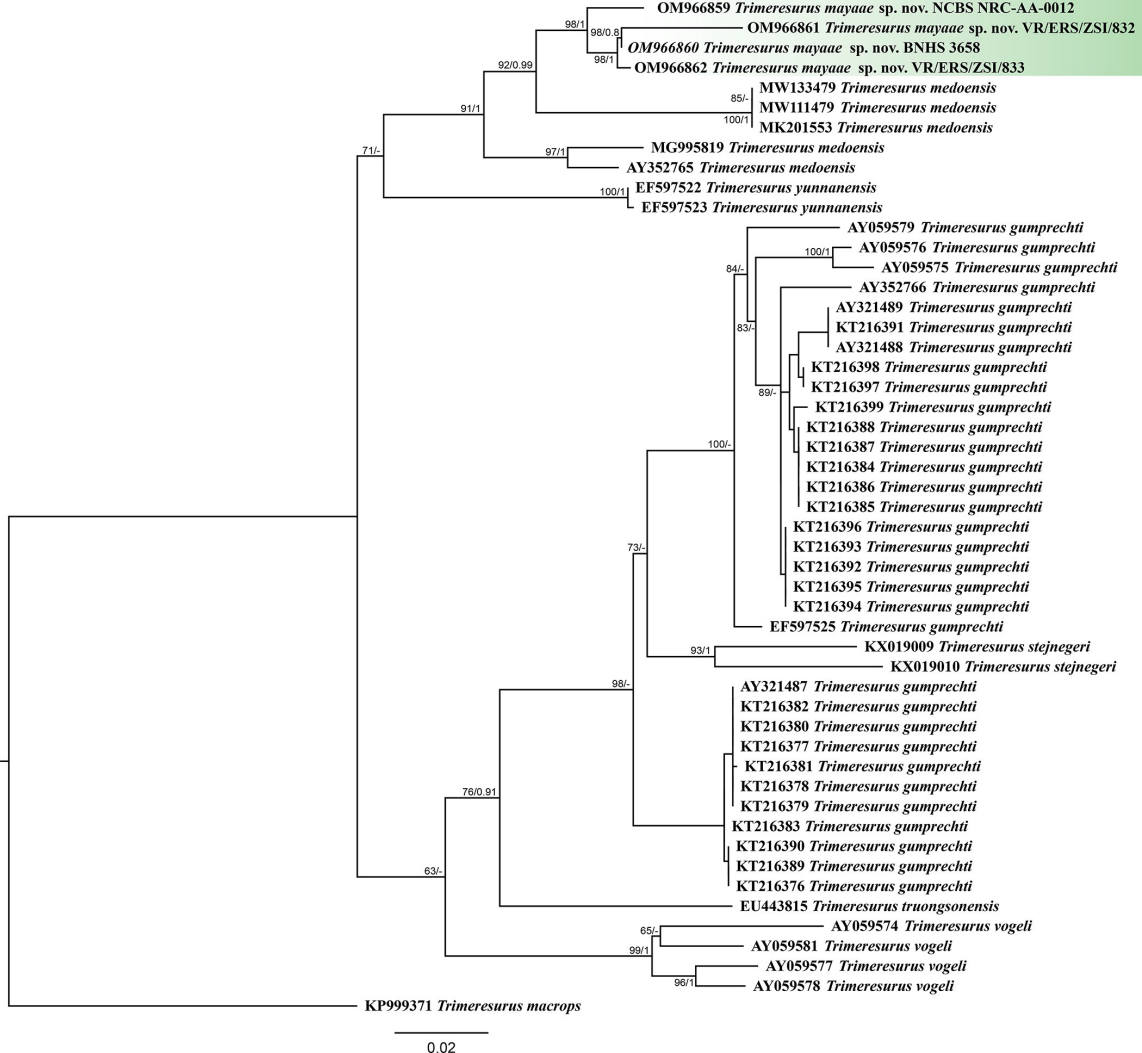
Maximum likelihood phylogeny of members of the subgenus *Viridovipera*. Numbers at nodes indicate ML bootstrap support through an ultra-fast search method. The outgroup has been remove, see S1 Fig for complete tree.

**Fig 2:**
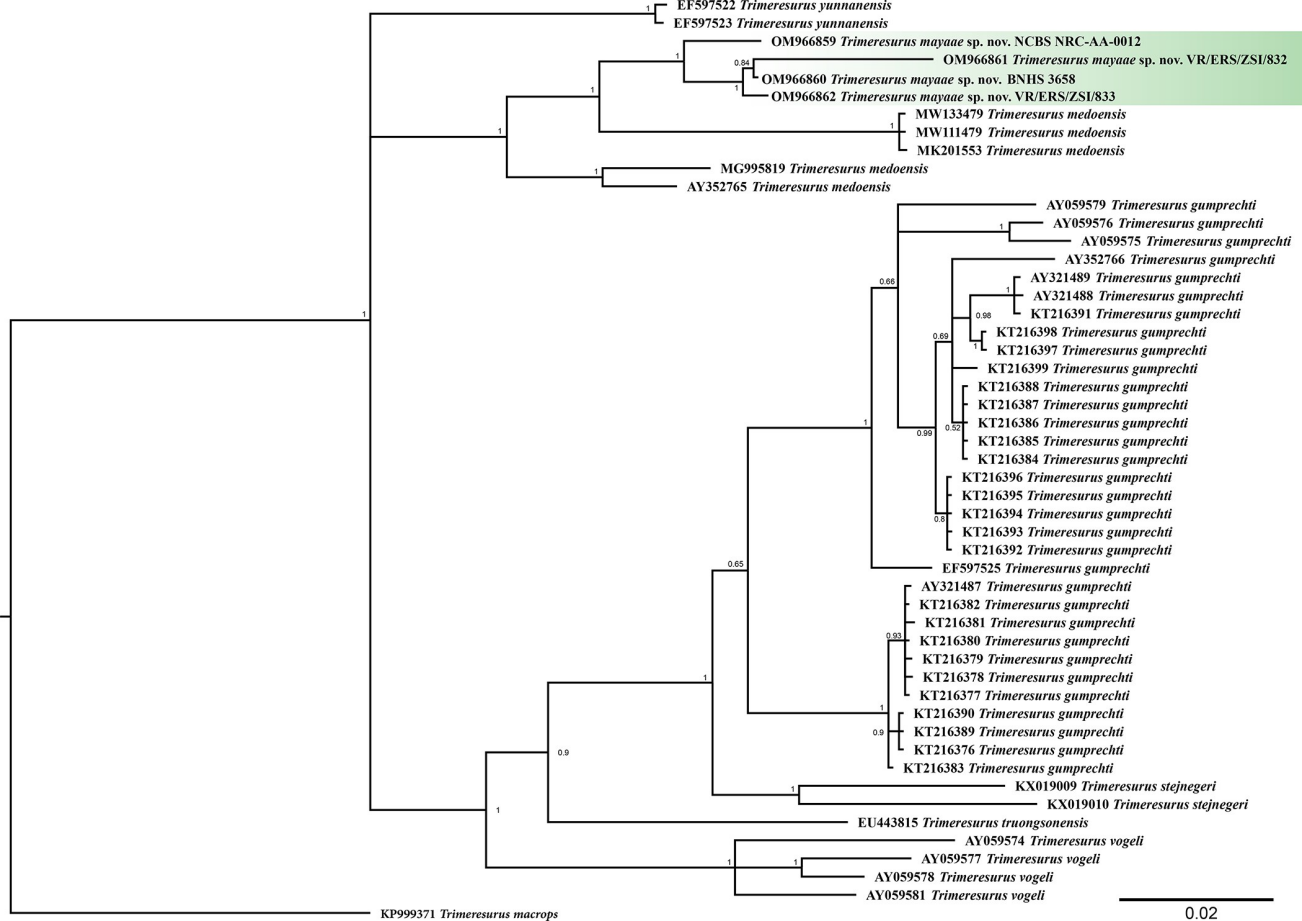
Bayesian inference phylogeny of members of the subgenus *Viridovipera*. Numbers at notes indicate BI posterior probability. The outgroup has been remove, see S2 Fig for complete tree.

The molecular phylogeny was based on 1080bp of *cyt b* gene for members of the subgenus *Viridovipera* and *Trimeresurus macrops* was used as an outgroup for the analysis. The ML analysis recovered two clades, one containing *Trimeresurus medoensis* and sequences of *Trimeresurus* from Mizoram and Meghalaya; the second clade contains *Trimeresurus gumprechti*, *Trimeresurus stejnegeri*, *Trimeresurus truongsinensis* and *Trimeresurus vogeli* (Fig 1). Relationship within the clade containing *Trimeresurus* from Mizoram and Meghalaya are well resolved and the *Trimeresurus* from Mizoram and Meghalaya is sister to *Trimeresurus medoensis* from China with high support (ML bootstrap 92). The BI analysis resulted in a slightly different tree topology with polytomy. *Trimeresurus gumprechti* was recovered monophyletic in BI (Fig 2) and paraphyletic in ML analysis. The discordance is likely due to different sequence evolution models (S2 Table) employed to inter phylogenetic relationships. However, despite the discordance in the approach in reconstructing phylogenetic relationships, the four sequences representing specimens from Mizoram and Meghalaya are genetically distinct from *Trimeresurus medoensis* and other members of the subgenus *Viridovipera* and is herein described as a new species with support from molecular as well as morphological data (see below).

### Systematics

#### *Trimeresurus mayaae* sp. nov.

*Trimeresurus stejnegeri* in part Malhotra & Thorpe 2004: 230

*Trimeresurus yunnanensis* in part Malhotra & Thorpe 2004: 230

*Trimeresurus gumprechti* David & Mathew 2005: 87

urn:lsid:zoobank.org:act: 8E1EF11A-0488-4AC0-B0FC-DE95E838FB15

Figs 3–5, Table 1

**Fig 3:**
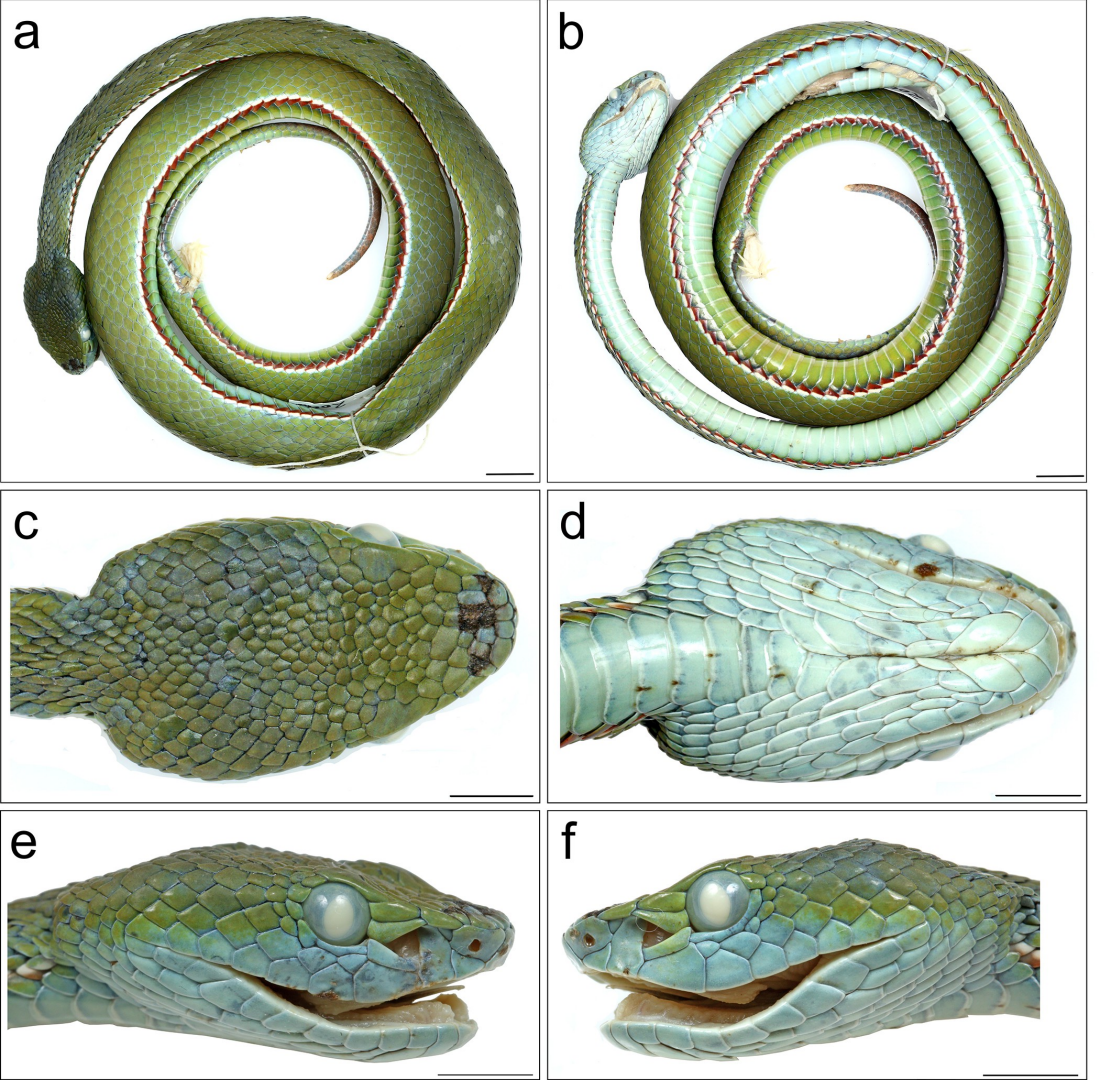
*Trimeresurus mayaae* sp. nov. holotype male NCBS NRC-AA-0012. (a) dorsal view, (b) ventral view, (c) head dorsal, (d) head ventral, (e) head lateral right, (f) head lateral left. Scale bar (a-b) 20mm, (c–f) 10mm. Photos by Zeeshan A. Mirza.

**Fig 4:**
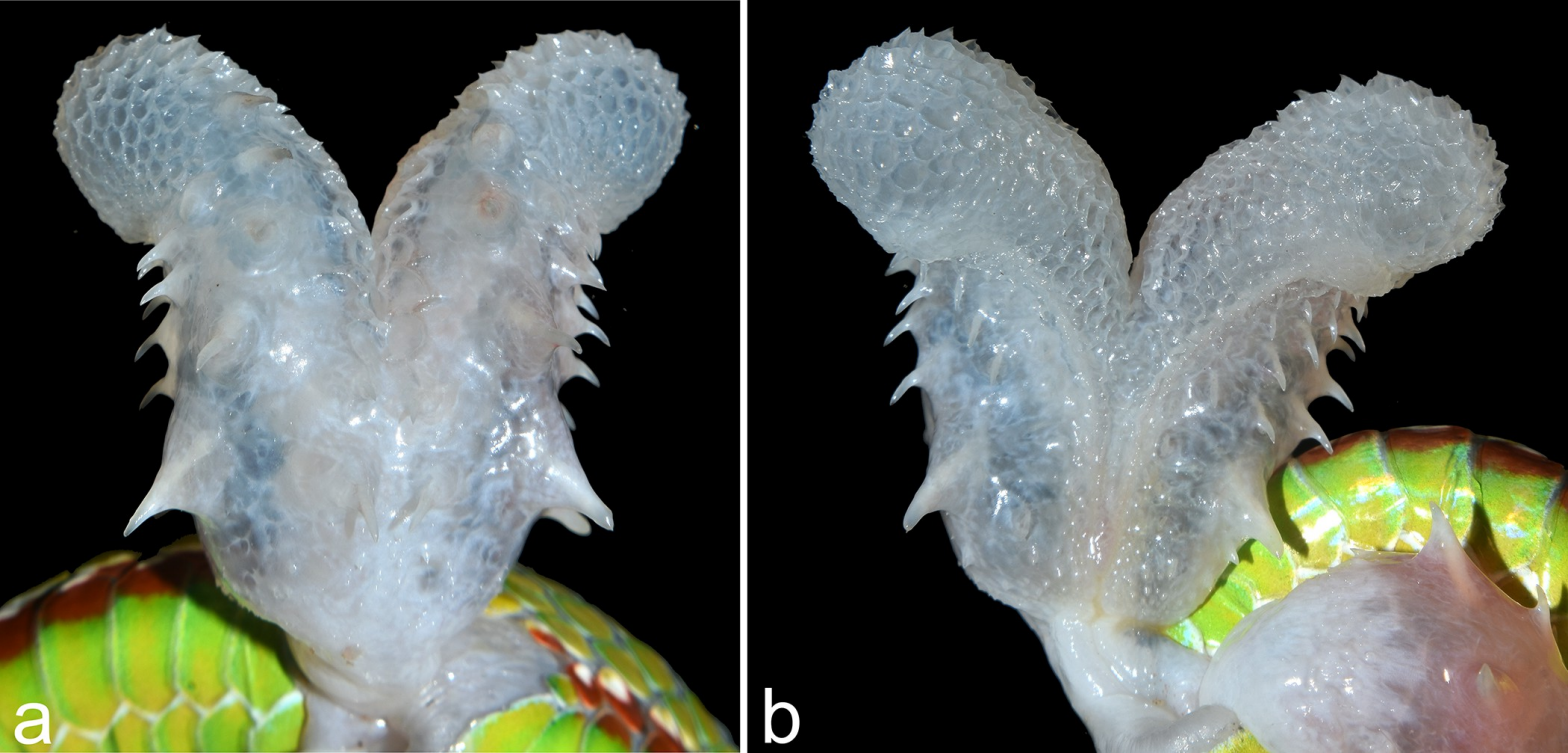
Hemipenis of paratype male BNHS 3658, (a) asulcal view, (b) sulcal view.

**Fig 5:**
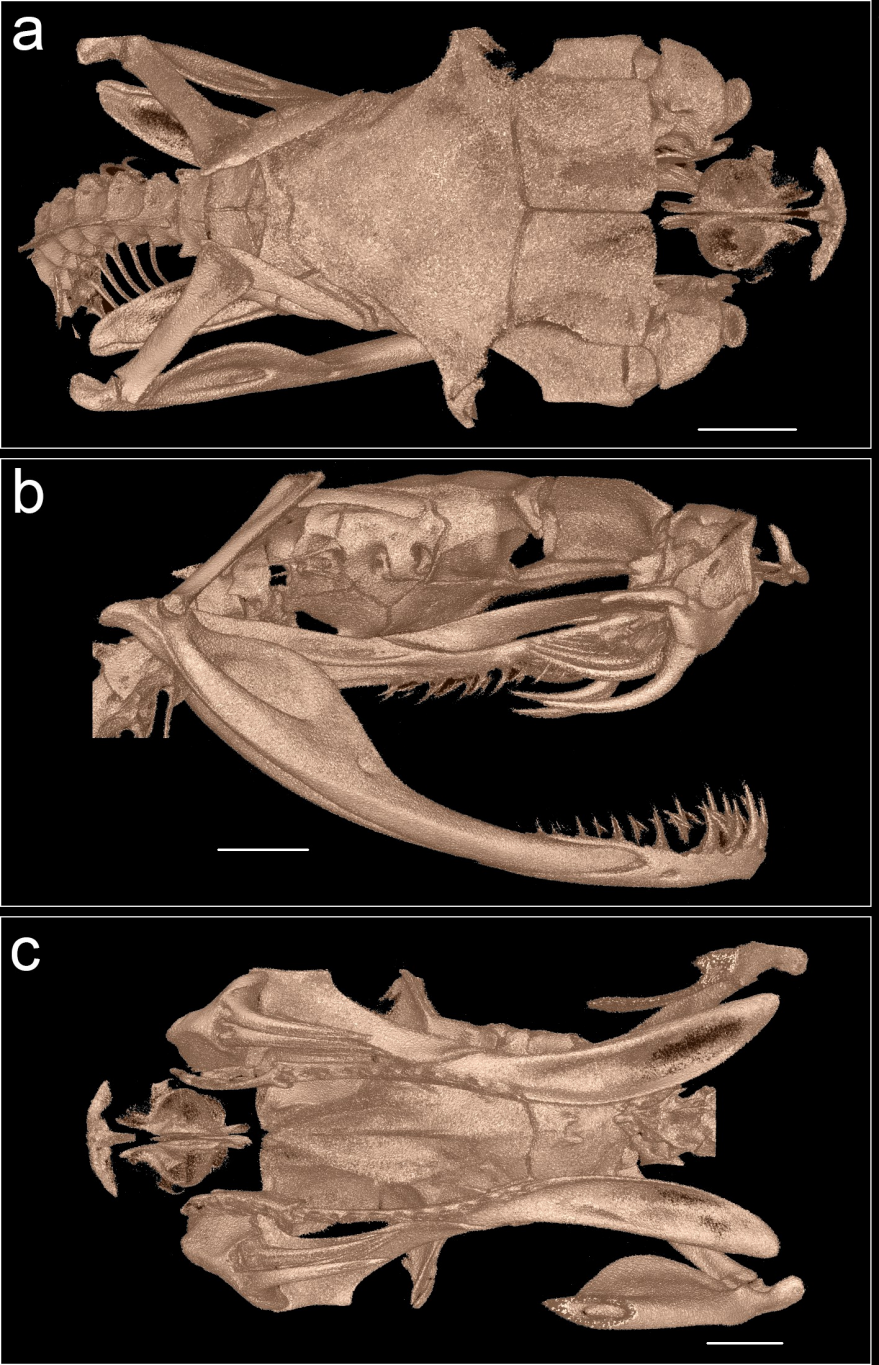
MicroCT images of the skull of male paratype BNHS 3660, (a) dorsal view, (b) lateral right view, (c) ventral view (dentary removed for visualizing the ventral aspect of the brain case).

**Table 1:** Meristic and morphometric data for the type series of *Trimeresurus mayaae* sp. nov.

Type status	Holotype	Paratype	Paratype	Paratype	Paratype	Paratype	Paratype
**Voucher number**	NCBS NRC-AA-0012	BNHS 3658	VR/ERS/ZSI/834	VR/ERS/ZSI/835	BNHS 3660	VR/ERS/ZSI/832	VR/ERS/ZSI/833
**Sex**	♂	♂	♂	♂	♂	♀	♀
**SVL**	490	610	460	450	520	555	590
**TaL**	150	140	80	35*	120	110	120
**TL**	640	750	540	485*	640	665	710
**TaL/TL**	0.23	0.19	0.15	-	0.19	0.17	0.17
**DSR**	21:19:17	28:21:15	25:20:15	21:21:15	21:21:15	21:21:16	25:21:15
**V (preventrals)**	157 (2)	160 (4)	160 (3)	162 (3)	162 (2)	153 (4)	153 (4)
**Sc**	54	67	57	25*	69	55	54
**A**	1	1	1	1	1	1	1
**SupraL**	9/9	10/9	8/9	9/9	10/10	10/9	10/9
**InfraL**	10/11	11/11	11/11	10/10	10/11	13/11	13/11

Values bearing ‘*’ indicate broken/damaged.

#### Holotype

Adult male NCBS NRC-AA-0012 (ex. MZMU 2043) from the ground in the homestead garden (23.4758959 N, 93.33401 E; altitude 1418 m a.s.l.), Bethel Veng, Champhai, Champhai District, Mizoram. Collected on 24 October 2020 by P.S. Muanga, H.T. Lalremsanga, Lal Muansanga and Lal Biakzuala.

#### Paratypes (n = 6)

Adult males, BNHS 3658 & BNHS 3660; two adult males, VR/ERS/ZSI/834 MZMU2730 (field number YRS005) and VR/ERS/ZSI/835 (field number YRS006), two adult female VR/ERS/ZSI/832 (field number YRS001) and VR/ERS/ZSI/833 collected from near a residential area, Umroi Military Station, Umroi, Ri-Bhoi District, Meghalaya (25.68193 N, 91.94471 E; elev. 930 m asl). Collected in the last two weeks of February 2021 by Yashpal Singh Rathee.

#### Referred material

ZSI/ERS 486 & BNHS 2634, Shillong, Meghalaya, India.

#### Diagnosis

A species of the genus *Trimeresurus*, characterized by (1) hemipenes short and strongly spinose; (2) body green in both males and females; (3) interstitial skin black; (4) moderate size, with maximum total length of 750mm; (5) conspicuous bicolored postocular stripe in males, thin and white below, wide and bright red above, faint white or no postocular stripe present in female (6) vivid, wide bicolored ventrolateral stripe, deep red below/white above in males, extending along the lower half of the tail, white in females; (7) eyes rust coloured in males, green in females; (8) tail mostly rusty or reddish-brown (9) V: 157–162; SC: 54–67 in males and V: 153; SC: 54–55 in females; (10) first supralabial distinct from nasal; (11) 19 or 21 dorsal scale rows at midbody, moderately keeled; (12) snout covered with rather enlarged juxtaposed scales; (13) internasals never in contact, separated by 1–2 scale; (14) supraoculars narrower than internasals, separated by 9–10 smooth cephalic scales.

#### Etymology

The species epithet is an eponym honouring late Maya Singh Rathee, mother of Yashpal Singh Rathee. English name: Maya’s pit viper.

#### Description of holotype male NCBS NRC-AA-0012

The specimen is in a good state of preservation, set in a loose coil bearing a longitudinal incision from 70^th^ to 80^th^ ventral (Fig 1A). One of the hemipenis is partly everted (Fig 3A). Body long (SVL 490 mm, TaL 150 mm, TaL/TL 0.23) and thin (BW 9.4); head moderately sized (HL/SVL 0.04) triangular, longer than broad (HW/HL 0.7) clearly distinct from neck; greatest internarial distance 4.4 mm and interorbital distance 9.7 mm. Distance between tip of snout to the anterior border of nostril is 1.5 mm, to the anterior border of the pit is 4.8 mm and to the anterior border of the eye is 8.3 mm; distance between posterior border of nostril to the anterior border of the pit is 2.5 mm and to the anterior border of the eye is 6.0 mm. Eye large (ED 3.8), circular with ED/ DEL ratio 1.3; Nostril eliptical about one-fifth of the eye diameter (Nostril diameter 0.8 mm). Canthus rostralis distinct; three scales between internasal and supraocular. Rostral subtriangular (2.9 mm wide, 2 mm high), slightly visible when viewed from above; nasal completely free from first supralabial, wider than high (2.9 mm high, 2 mm wide); a pair of subrectangular internasals separated from each other by two scale. The loreal pit is subtriangular in shape and the greatest length of the pit is 1.8 mm; second supralabial and two preoculars encompass the loreal pit; the lower preocular forms the lower margin of the loreal pit (Fig 3A and 3B); one elongate supraocular (2.4 mm wide, 4.6 mm long); cephalic scales (CEP) larger anteriorly with the largest scales between the internasal and supraocular, subimbricate, smooth; LCS 26, more elongate near the neck; 9 CEP between supraoculars (Fig 3D); occipital scales smooth; three rows of scales between the internasals and anterior border of the supraoculars; subocular crescent shaped; 9/9 supralabials; 1st supralabial triangular, 2^nd^ 1.9 times higher than broad (H: 3.4mm, W: 1.7mm); 3rd supralabial is the widest amongst all supralabials (4 mm) and is in contact with 2^nd^ and 4^th^ supralabials, lower preocular and a small scale between subocular and supralabials; 4th supralabial small, separated from the subocular by a single row of smooth scales; 5th supralabial in contact with temporal; the remaining supralabials slightly decreasing in size posteriorly and in contact with temporal scales; 10/11 infralabials, the first pair in contact with each other; the first three pairs in contact with anterior chin shields; six pairs of chin shields, 1^st^, 2^nd^, 5^th^ and 6^th^ contact medially, 3^rd^ and 4^th^ separated by a pair and a single small scale respectively; separated from infalabials by 1–5 scale rows (Fig 2C).

Body scalation: 21 dorsal scales one head length behind the head; 19 dorsal scales at midbody; 17 dorsal scales one head length anterior to the vent; dorsal scales rhomboid, moderately keeled except for the first row, which is smooth; two preventrals; 157 ventral scales;, 54 subcaudal scales;, paired; anal shield entire. tail short; ventrally depressed; Tal 150 mm; TaL/TL 0.19. Tail prehensile.

Hemipenis based on paratype BNHS 3658 (Fig 4): Hemipenis short, bilobed, not deeply forked, extending to the ninth caudal scale. The total length of the hemipenis is 24.3 mm and the greatest width is 15.5 mm. From the base, the hemipenis gets birfurcated at around half of its length (12.8 mm). Four enlarged spines, two at each lateral side of the hemipenis are seen at one-fourth length from the base. Many enlarged spines are also at the lateral surface of the hemipenis extending upto the region of bifurcation. The bifurcated section of the hemipenis are without spines and resembles honeycomb lattice. Sulcus spermaticus at around one-fourth of length of the hemipenis from the base and extends up to the bifurcated section of the Hemipenis.

Skull of paratype male BNHS 3660 (Fig 5): The skull is longer than wide in dorsal view. The premaxilla, nasal and septomaxilla appear unattached to other sets of bones in the dorsal view. The palatine is crescent-shaped similar to that of *T*. *stejnegeri* [27] and is unarticulated at its anterior end, and bears 3–4 teeth. On its posterior end, it is articulated with the pterygoid. The maxilla bears one functional and 4–7 replacement fangs. The pterygoid is long and slender at its anterior end with 10 teeth until the middle of the bone. The posterior part extends well beyond the quadrate and dentary bone articulation and is visible in the dorsal view. The dentary bears 10–12 functional teeth; the anterior ones are larger, which gradually decreases in size towards the posterior part. The basioccipital bears a pair of low processes.

**Fig 6:**
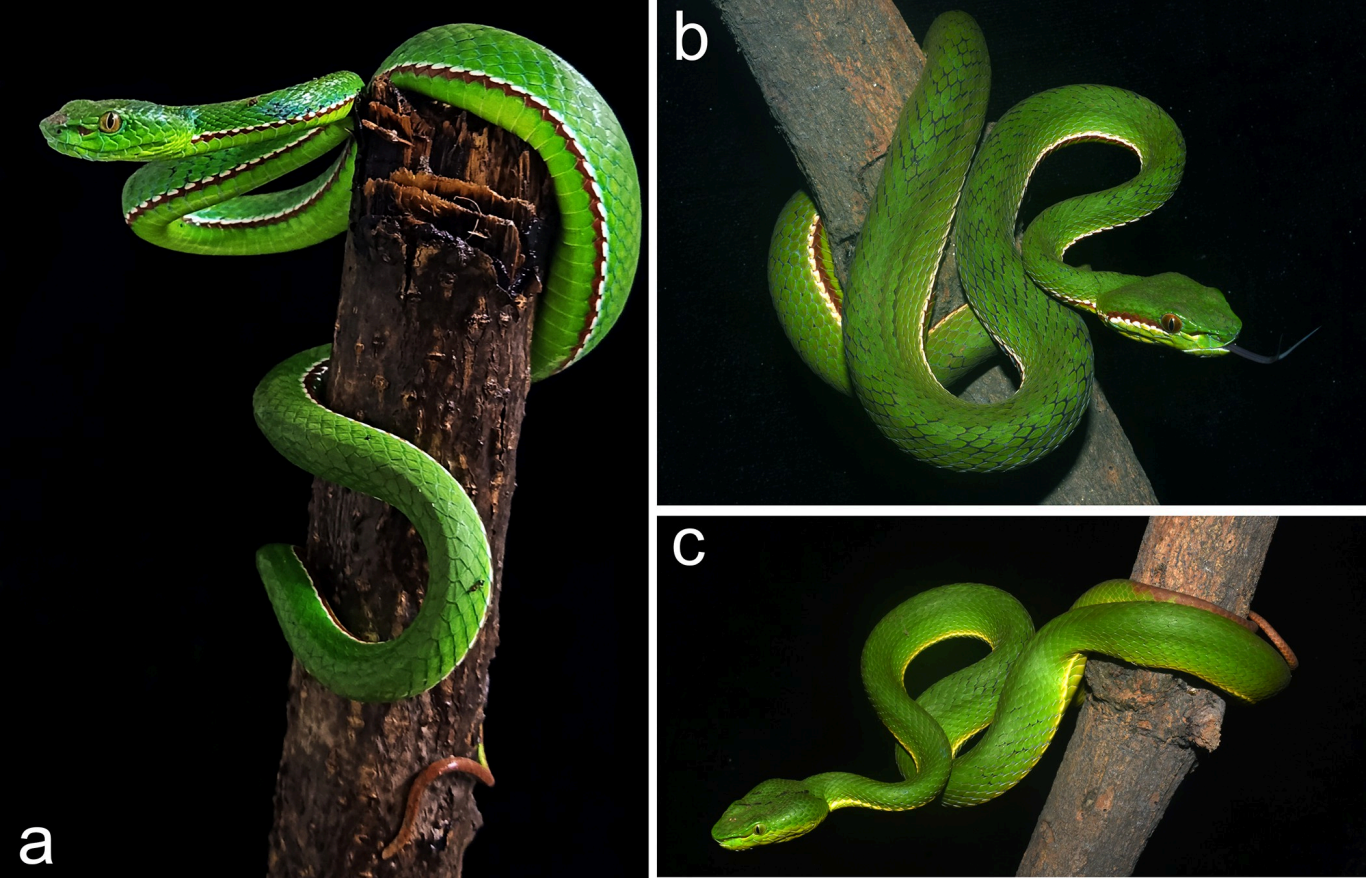
*Trimeresurus mayaae* **sp. nov.** in life (a) holotype male NCBS NRC-AA-0012, Photo Hmar Tlawmte Lalremsanga, (b) uncollected male photo by Hmar Tlawmte Lalremsanga, (c) uncollected female photo by Jayaditya Purkayastha.

Colouration in life (Fig 6): Males. Overall dorsum in a shade of deep green, ventrum fluorescent green. Interstitial skin black. Dorsally head is dark green. The green colouration fades to a lighter shade towards the lateral portion of the head. Each green scale is edged with cyan and the cyan bordering increases on each scale, especially on the scales on the lateral and ventral aspect of the head. The cyan colouration covers most areas of the scale concealing the green colouration within and may appear as cyan speckles on the green area. Eyes rust coloured. A bicoilored (broad and red above, thin and white below) strip runs from behind the eyes until the angle of the jaw. The post ocular stripe is 2–3 rows of scales in width. The ventrolateral stripe is bicoloured (white above and red below) and is one scale thick, with the red colour dispersing on to the edge of the adjacent ventral scale. The ventrolateral stripe runs almost to the half of the tail. More than half of the posterior part of the tail, rusty red. Females, dorsum green, ventrum yellowish green postocular stripe faint, white or absent. Eyes green coloured. A yellowish white ventrolateral stripe present. More than half of the posterior part of the tail, rusty red.

Colouration in preservative (Fig 3): All the colouration fades away with the entire body becoming bluish green in colour. The paratypes have turned brown likely an artifact of preservation.

#### Variation

Refer to Table 1 for morphometric and basic pholidosis variation within the type series of *Trimeresurus mayaae* **sp. nov.**, comprising one adult male, two sub-adult males and two adult females. In most cases, the paratype agrees to the description of the holotype. The only female differs in coloration from the males as described in the colouration section. The male paratypes bear a distinct postocular stripe, which the male holotype lacks. Specimen MZMU2730 has 20 scales in the mid-body rather than 21. The variation in the scale rows at mid-body is not sex linked but likely the variability in collecting data by the observer.

### Comparison

Based on the molecular data and hemipenis structure, *Trimeresurus mayaae* **sp. nov.** is found to be a member of subgenera *Viridovipera*. The species differs from all the known members of *Viridovipera* by following characters (Table 2):

Morphologically and meristically *Trimeresurus mayaae* **sp. nov.** closely resembles *T*. *gumprechti*, but differs from the latter by having rust coloured or greenish eye in males versus bright or deep red in *T*. *gumprechti* and green coloured eye in female versus golden yellow in *T*. *gumprechti*. Furthermore, *Trimeresurus mayaae* **sp. nov.** has 1–2 scales between internasal versus 0–1 in *T*. *gumprechti*.; ventral scales range 153–162 in *Trimeresurus mayaae* **sp. nov.** versus 162–168 *T*. *gumprechti*; subcaudals 54–69 in *Trimeresurus mayaae* **sp. nov.** versus 51–71 in *T*. *gumprechti*; TaL/TL in females 0.17 in *Trimeresurus mayaae* **sp. nov.** versus 0.14–0.16 in *T*. *gumprechti*. *Trimeresurus mayaa****e* sp. nov.** differs from all the other members of subgenera *Viridovipera* by having rust coloured eye in males versus bright red or amber (rarely yellow) coloured in *T*. *stejnegeri* Schmidt, yellow or yellowish green in *T*. *vogeli* David, Vidal & Pauwels, green or yellowish green in *T*. *medoensis* Zhao, and bright or deep red in *T*. *yunnanensis*, and a green coloured eye in females versus yellow or amber in *T*. *stejnegeri*, yellow in *T*. *vogeli* and golden yellow in *T*. *yunnanensis*. A bicoloured (broad red above and thin white below) postocular streak is present in males of *Trimeresurus mayaae* **sp. nov.** versus white in *T*. *stejnegeri*, white, thin and faint in *T*. *vogeli*, no postocular streak in *T*. *yunnanensis*. A white ventrolateral stripe is present in female *Trimeresurus mayaae* **sp. nov.** versus red + white in *T*. *medoensis* and pale green in *T*. *yunnanensis*. *Trimeresurus mayaae* **sp. nov.** has 19–21 dorsal scale rows at the midbody versus 17 in *T*. *medoensis* and 19 in *T*. *yunnanensis*. *Trimeresurus mayaae* **sp. nov.** with 3–4 palatine teeth versus 5 in *T*. *yunnanensis*; *Trimeresurus mayaae* **sp. nov.** with 10 pterygoid teeth versus 12–14 in *T*. *yunnanensis* & *T*. *stejnegeri*. The new species differs from members of *Viridovipera* in showing a pairwise sequence divergence (p-distance) of 5–10% for cytochrome b gene (S1 Table).

**Table 2:** A summary of characters of members of the subgenus *Viridovipera*. Data derived from Gumprecth et al. [9].

Species	Eye colour	Post-ocular stripe	Body colouration	Dorsal Scale rows	Ventrals	Subcaudals
*T*. *mayaae* sp. nov.	rust coloured or greenish	red and white (may be absent)	bright green without bands or markings	21,25,28:19, 20, 21:15, 16, 17	157–162 ♂	54–69 ♂
	greenish				153 ♀	54–55 ♀
*T*. *gumprechti*	bright or deep red	red and white	bright green without bands or markings	21–23(25):21:15	162–168 ♂	55–71 ♂
	golden yellow				163–170 ♀	51–60 ♀
*T*. *medoensis*	green	absent	bright green without bands or markings	17:19(21):17:13(15)	138–149 ♂	54–65 ♂
					141–149 ♀	52–60 ♀
*T*. *stejnegeri*	bright red or amber (rarely yellow)	white	bright green without bands or markings	21–23(22,24,25):21:15(12,13)	154–170 ♂	60–75 ♂
					154–172 ♀	43–73 ♀
*T*. *vogeli*	yellow or yellowish green	faint and thin white	bright green without bands or markings	21–23:21:15	163–173 ♂	62–72 ♂
					167–163 ♀	58–64 ♀
*T*. *yunnanensis*	bright or deep red	absent	bright green without bands or markings	19(20, 21):19:15	154–164 ♂	61–71 ♂
					150–172 ♀	52–65 ♀
*T*. *truongsonensis*	greenish-yellow	absent	Greenish blue with brown broad bands males	-:21:15	166–176 ♂	65–69 ♂

*Trimeresurus mayaae* **sp. nov.** differs from other species of *Trimeresurus* occurring in northeast India by following characteristics:

*Trimeresurus mayaae* **sp. nov.** differs from *T*. *albolabris*, *T*. *arunachalensis* Captain, Deepak, Pandit, Bhatt & Athreya, *T*. *erythrurus*, *T*. *septentrionalis* Kramer, *T*. *salazar* , *T*. *popeiorum* Smith, by having a short spinose hemipenis versus long papillose hemipenis without spines; first supralabial completely free from nasal versus first supralabial partially or completely fused with nasal (except for *T*. *popeiorum* which has first supralabial completely free from nasal). *Trimeresurus mayaae* **sp. nov.** has 19–21 mid-dorsal scale rows versus 17 in *T*. *arunachalensis*; 23–25 in *T*. *erythrurus*.

*Trimeresurus mayaae* **sp. nov.** differs from all the other species of *Trimeresurus* by a combination of following characteristics:

*Trimeresurus mayaae* **sp. nov.** has a short spinose hemipenis versus a long papillose hemipenis without spines in *T*. *fasciatus* (Boulenger), *T*. *flavomaculatus* (Gray), *T*. *gunaleni* Vogel, David & Sidik. *T*. *guoi* Chen, Shi, Vogel & Ding, *T*. *insularis* Kramer, *T*. *kanburiensis* Smith, *T*. *macrops* Kramer, *T*. *mcgregori* Taylor, *T*. *nebularis* Vogel, David & Pauwels, *T*. *phuketensis* Sumontha, Kunya, Pauwels, Nitikul & Punnadee, *Trimeresurus sabahi* Regenass & Kramer, *T*. *sumatranus* (Raffles), *T*. *tibetanus* Huang, *T*. *venustus* Vogel, *T*. *yingjiangensis* Chen, Zhang, Shi, Tang, Guo, Song & Ding.

*Trimeresurus mayaae* **sp. nov.** has 19–21 mid-dorsal scale rows versus greater than 23 scale rows in *T*. *andersonii* Theobald, *T*. *cantori* (Blyth), *T*. *gracilis* Oshima, *T*. *purpureomaculatus* (Gray) and less than 19 scale rows in *T*. *kanburiensis*, *T*. *macrolepis* Beddome, *T*. *malcolmi* Loveridge, *T*. *trigonocephalus* (Latreille).

In *Trimeresurus mayaae* **sp. nov.** first supralabial is completely free from nasal versus first supralabial partially or completely fused with nasal *in T*. *andersonii*, *T*. *cantor*, *T*. *cardamomensis* (Malhotra, Thorpe, Mrinalini & Stuart), *T*. *davidi* Chandramouli, Campbell & Vogel, *T*. *hageni* (Lidth De Jeude), *T*. *labialis* (Steindachner), *T*. *purpureomaculatus*, *T*. *rubeus* (Malhotra, Thorpe, Mrinalini & Stuart)

*Trimeresurus mayaae* **sp. nov.** has an immaculate green dorsal colouration versus dorsal colouration with blotches, spots, bands or crossbars in *T*. *andalasensis* David, Vogel, Vijayakumar & Vidal, *T*. *andersonii*, *T*. *borneensis* (Peters), *T*. *brongersmai* Hoge, *T*. *cantori*, *T*. *fasciatus*, *T*. *flavomaculatus*, *T*. *gracilis*, *T*. *gramineus* (Shaw), *T*. *gunaleni*, *T*. *guoi*, *T*. *hageni*, *T*. *honsonensis* (Grismer, Ngo & Grismer), *T*. *kanburiensis*, *T*. *labialis*, *T*. *malabaricus* (Jerdon), *T*. *mcgregori*, *T*. *mutabilis* Stoliczka, *T*. *puniceus* (Boie), *T*. *purpureomaculatus*, *T*. *schultzei* Griffin, *T*. *strigatus* Gray, *T*. *sumatranus*, *T*. *tibetanus*, *T*. *trigonocephalus*, *T*. *truongsonensis* Orlov, Ryabov, Thanh & Cuc, *T*. *venustus*, *T*. *wiroti* Trutnau.

*Trimeresurus mayaae* **sp. nov.** has a bicoloured (red above white below) postorbital stripe versus postorbital stripe absent in *T*. *caudornatus* Chen, Ding, Vogel & Shi and *T*. *sichuanensis* (Guo & Wang).

### Natural history

The holotype was collected at dusk at around 17:15h. The specimen was found on the ground in the homestead garden planted with *Emblica officinalis*, *Mangifera indica*, *Psidium guajava*, *Prunus domesticus*, etc. at Bethel Veng, Champhai, a district capital of Champhai District, Mizoram. According to Champion and Seth [28], the area falls under the Assam Subtropical Pine Forest (*9/C2*) which is mostly dominated by *Pinus kesiya* with ether associates like *Quercus spp*., *Schima wallichii*, *Rhododendron spp*., *Castanopsis spp*., *Lyonia ovalifolia*, *Rhus spp*., *Myrica esculenta*, *Prunus* spp. etc. Champhai has a moderate climate. In winter, the temperature varies from 10°C to 20°C and between 15°C and 30°C in summer, with 2037.6 mm annual rainfall. All the paratypes were found during early hours of night in a forested patch next to a stream within Umroi Military Cantonment area. An individual was spotted on the ground, crossing a track. In captivity, the specimens fed on the *Rhacophorus bipunctatus*, *Leptobrachium* sp. and *Minervarya* sp.

## Discussion

**Fig 7:**
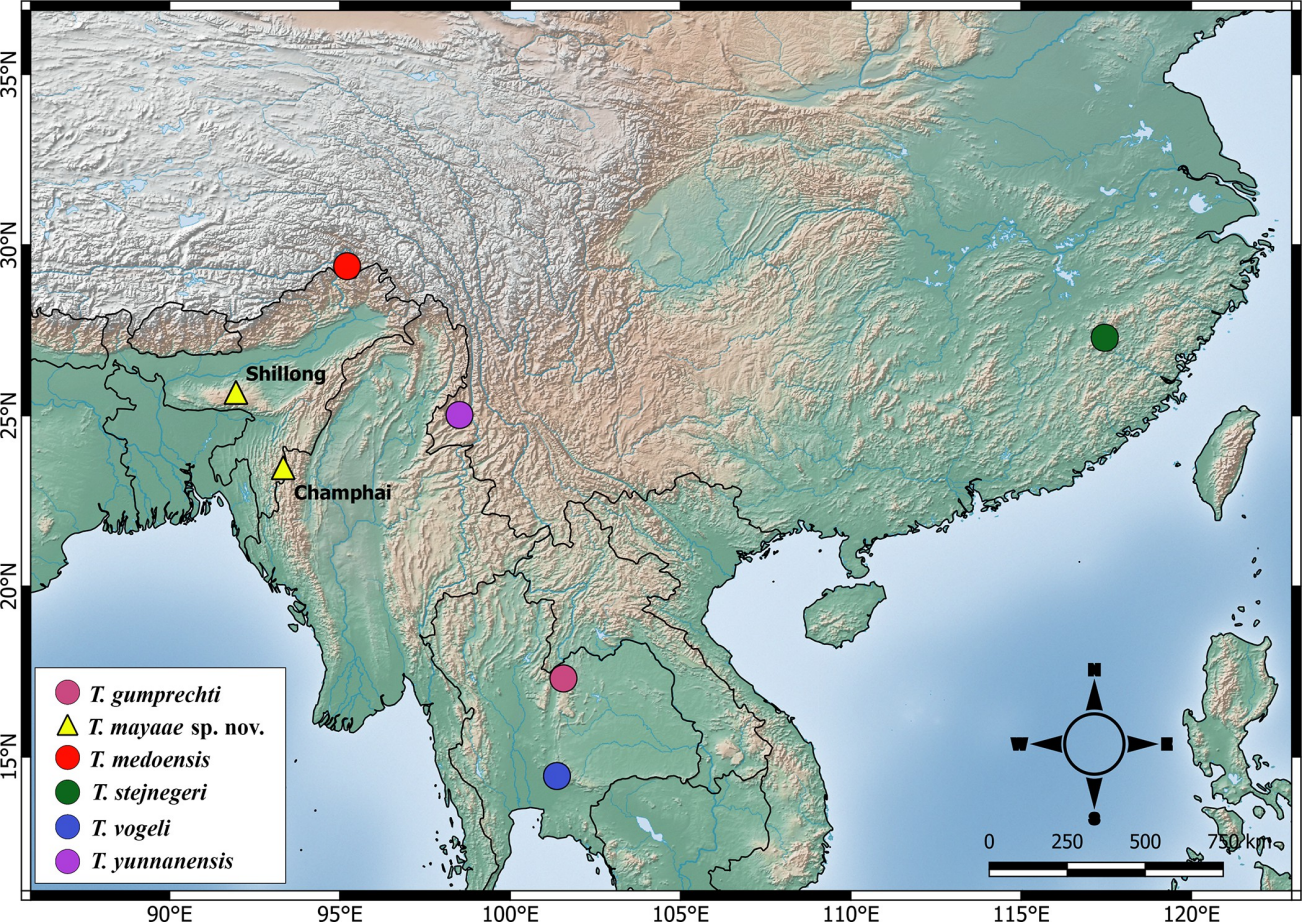
Map showing distribution of the new species and type localities of members of the subgenus *Viridovipera*.

Molecular data for partial fragments of *cyt* b suggests that the new species is a member of the subgenus *Viridovipera*. Based on 1080bp of *cyt* b gene, the new species is sister to *T*. *medoensis s*. *l*.. Further work with data for additional genes is necessary to resolve the phylogenetic relationship of the new species with other members of the subgenus because of unresolved relationships in BI. The new species shows an intraspecific divergence of 0–4% and an interspecific divergence of 5.2–10% from members of the subgenus *Viridovipera* (S1 Table). The new species occurs throughout the Shillong Plateau and the adjoining Jaintia hills, Barail and Mizo hills. This area is isolated from other members of *Viridovipera*, by low elevation human dominated landscape, Brahmaputra River to its north and the Indo-Burma hills along the international borders of the two countries (Fig 7). All known records of the new species are from elevations >900m and hence, the low land areas may potentially act as a biogeographic barrier for the new species. Brahmaputra River is a known barrier for several terrestrial tetrapod [29], which further separates the new species from its geographically and genetically proximate relative, *T*. *medoensis* in the north.

Molecular data for green pit vipers from throughout their range hints on presence of morphologically cryptic species [30–33]. Lack of molecular data for *Trimeresurus* from northeast India has led to misidentification of most taxa [2]. Similarly, David and Mathew [13] identified the new species described herein as *T*. *gumprechti*. David and Mathew [13] considered the records of *T*. *yunnanensis* and *T*. *stejnegeri* from northeast India [30] to be of T. *gumprechti*. Description of the new species, which was identified as *T*. *gumprechti* (ZSI/ERS 486) suggests that the records of *T*. *yunnanensis*, *T*. *stejnegeri* and *T*. *gumprechti* represent the new species described herein.

Recent investigations on the systematics of pit vipers of northeast India have led to the discovery of three new species, including the present work [2, 34]. An appraisal of *Trimeresurus* from across India would be necessary to ascertain the exact diversity within the genus and its biogeography. Molecular data for *T*. *medoensis s*. *l*. suggests presence of distinct lineages across sampled areas of Indo-China, so does *T*. *gumprechti*. Sampling for pit vipers from across Northeast India, especially of widespread species like *Trimeresurus popeiorum*, *Trimeresurus albolabris* and *Trimeresurus erythrurus*, will help evaluate if there are cryptic species within broadly distributed and morphologically cryptic groups. Northeast India is rich in its reptile diversity, which still remains poorly studied. Several new species have been described in the recent past [34–38]. Many road widening and hydropower plant projects are some of the few anthropogenic pressures to the forest and species across northeast India with regards to their conservation.

## Supporting information

S1 Fig Maximum likelihood phylogeny of members of the subgenus *Viridovipera*. Numbers at nodes indicate ML bootstrap support through an ultra-fast search method.
(PDF)

S2 Fig Bayesian inference phylogeny of members of the subgenus *Viridovipera*. Numbers at notes indicate BI posterior probability.
(PDF)

S1 Table Un-corrected pairwise sequence divergence on *cyt* b gene for members of the subgenus *Viridovipera*. (XLS)

S2 Table Sequence evolution model used in ML and BI phylogenetic analysis. (XLSX)
