# Supplementary figures and images for: A new cryptic species of green pit viper of the genus Trimeresurus Lacépède, 1804 (Serpentes, Viperidae) from northeast India
Source: PLoS One. 2022 May 20;17(5):e0268402. doi: 10.1371/journal.pone.0268402 (PMC9122190; doi:10.1371/journal.pone.0268402)

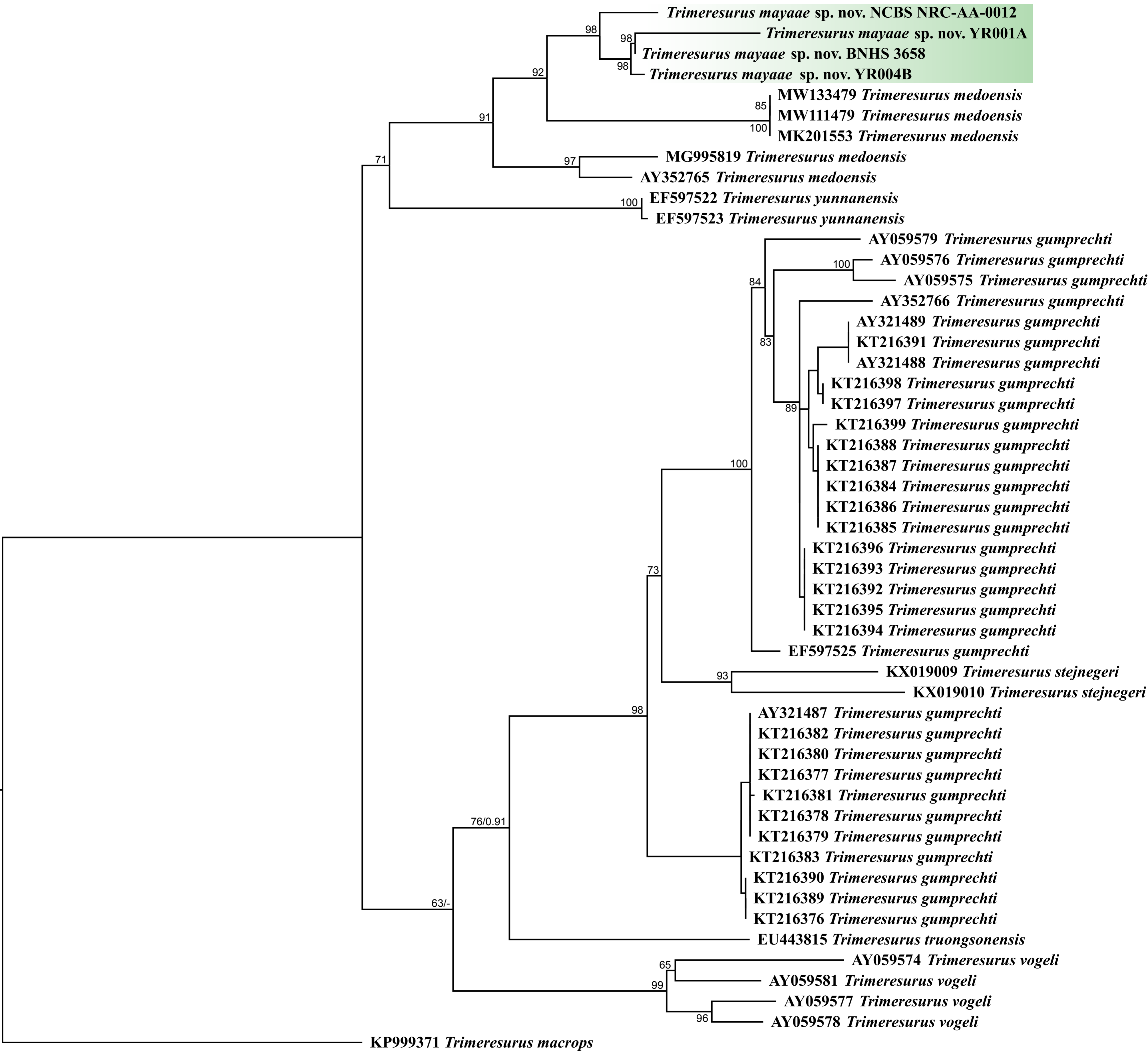

0.02

Supplement: S1 Fig — Numbers at nodes indicate ML bootstrap support through an ultra-fast search method. (PDF) [file pone.0268402.s001.pdf]

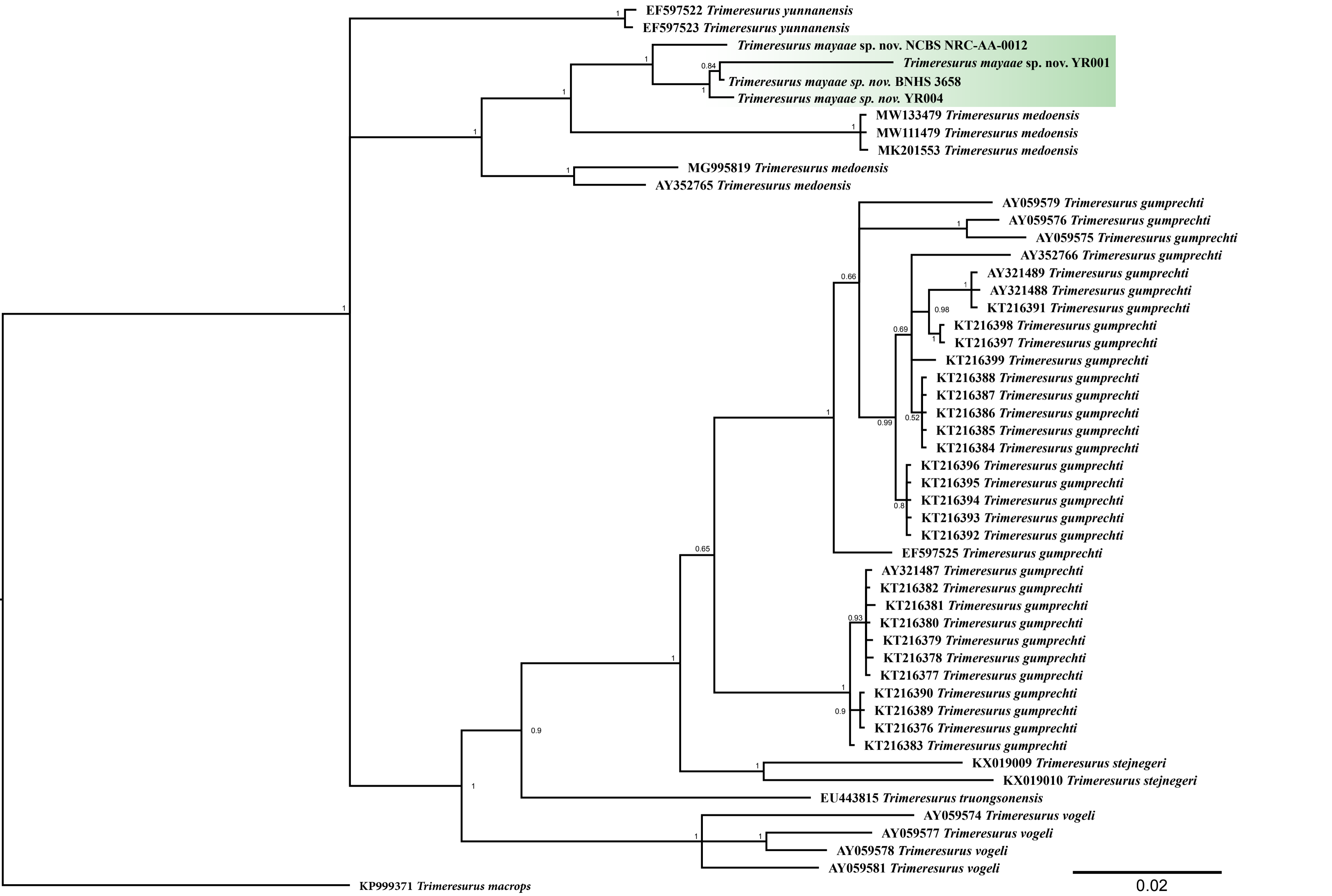

Supplement: S2 Fig — Numbers at notes indicate BI posterior probability. (PDF) [file pone.0268402.s002.pdf]
